# The impact of mass drug administration on* Schistosoma haematobium* infection*:* what is required to achieve morbidity control and elimination?

**DOI:** 10.1186/s13071-020-04409-3

**Published:** 2020-11-18

**Authors:** Klodeta Kura, Robert J. Hardwick, James E. Truscott, Jaspreet Toor, T. Deirdre Hollingsworth, Roy M. Anderson

**Affiliations:** 1 London Centre for Neglected Tropical Disease Research, London, UK; 2 Department of Infectious Disease Epidemiology, School of Public Health, Faculty of Medicine, St Mary’s Campus, Imperial College London, London, UK; 3 MRC Centre for Global Infectious Disease Analysis, London, UK; 4 The DeWorm3 Project, The Natural History Museum of London, London, UK; 5 Big Data Institute, Li Ka Shing Centre for Health Information and Discovery, University of Oxford, Oxford, OX3 7LF UK

**Keywords:** Schistosomiasis, Modelling, Mass drug administration, School-based treatment, Community-wide treatment, Morbidity control, Elimination as a public health problem

## Abstract

**Background:**

Schistosomiasis remains an endemic parasitic disease causing much morbidity and, in some cases, mortality. The World Health Organization (WHO) has outlined strategies and goals to combat the burden of disease caused by schistosomiasis. The first goal is morbidity control, which is defined by achieving less than 5% prevalence of heavy intensity infection in school-aged children (SAC). The second goal is elimination as a public health problem (EPHP), achieved when the prevalence of heavy intensity infection in SAC is reduced to less than 1%.

Mass drug administration (MDA) of praziquantel is the main strategy for control. However, there is limited availability of praziquantel, particularly in Africa where there is high prevalence of infection. It is therefore important to explore whether the WHO goals can be achieved using the current guidelines for treatment based on targeting SAC and, in some cases, adults.

Previous modelling work has largely focused on *Schistosoma mansoni*, which in advance cases can cause liver and spleen enlargement. There has been much less modelling of the transmission of *Schistosoma haematobium,* which in severe cases can cause kidney damage and bladder cancer. This lack of modelling has largely been driven by limited data availability and challenges in interpreting these data.

**Results:**

In this paper, using an individual-based stochastic model and age-intensity profiles of *S. haematobium* from two different communities, we calculate the probability of achieving the morbidity and EPHP goals within 15 years of treatment under the current WHO treatment guidelines. We find that targeting SAC only can achieve the morbidity goal for all transmission settings, regardless of the burden of infection in adults. The EPHP goal can be achieved in low transmission settings, but in some moderate to high settings community-wide treatment is needed.

**Conclusions:**

We show that the key determinants of achieving the WHO goals are the precise form of the age-intensity of infection profile and the baseline SAC prevalence. Additionally, we find that the higher the burden of infection in adults, the higher the chances that adults need to be included in the treatment programme to achieve EPHP.

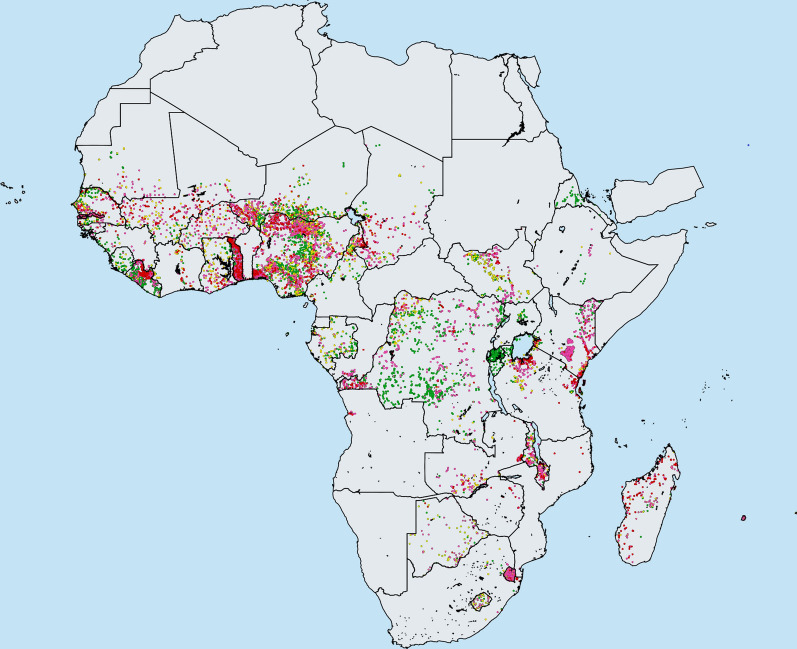

## Background

**Fig. 1:**
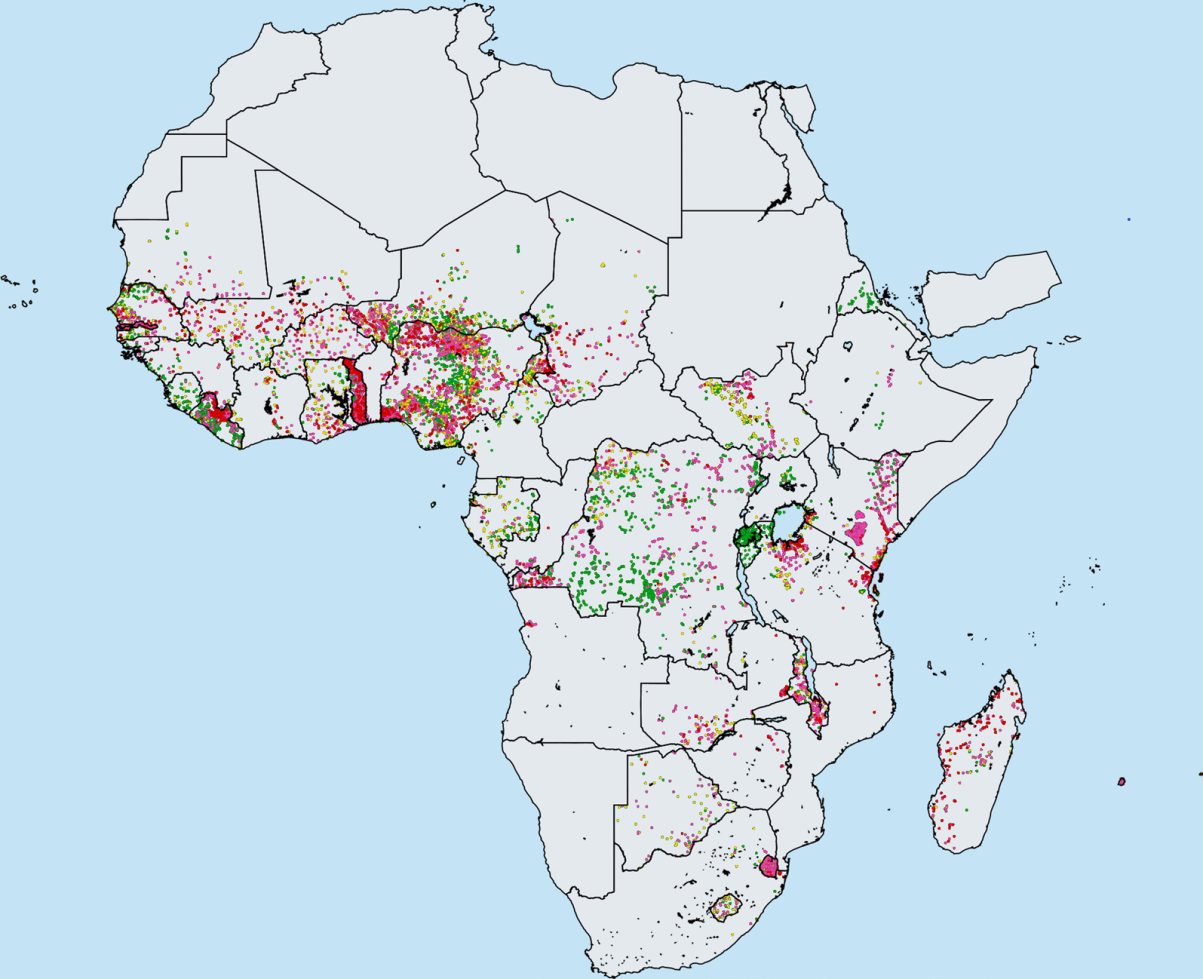
Distribution of *S. haematobium* in Africa in 2018 as reported by the Expanded Special Project for Elimination of Neglected Tropical Diseases (ESPEN). *Key*: green, prevalence < 1%; yellow, prevalence 1–9.9%; purple, prevalence 10–49.9%; red, prevalence ≥ 50% [24]

Schistosomiasis is a parasitic disease which affects nearly 229 million people worldwide, mostly in Africa (Fig. 1) [1]. The helminth parasites may mature to adult worms in either the intestines (the species *Schistosoma mansoni* or *S. japonicum*) or the urogenital tract (*S. haematobium*) [2]. The World Health Organization (WHO) has published guidelines to combat the morbidity and mortality induced by infection, which are defined as when the prevalence of heavy intensity infection in school-aged children (SAC; 5–14 years of age) is less than 5% and 1%, respectively [3]. Heavy-intensity infection (≥ 400 epg for intestinal schistosomiasis and > 50 eggs per 10 ml for urogenital schistosomiasis) can be detected using the Kato-Katz technique and urine filtration [4]. The end goal of the WHO control strategy is true elimination (interruption of transmission), which is achieved when the incidence of new infections in a community is reduced to zero [3].

Mass drug administration (MDA) employing praziquantel (PZQ) is the main form of control. WHO defines both the treatment frequency and coverage levels to be dependent on the prevalence of infection in a defined location. The current treatment recommendations are treatment of SAC once a year in high prevalence settings (≥ 50% baseline prevalence among SAC), once every 2 years in moderate prevalence settings (10–50% baseline prevalence among SAC) and once every 3 years in low prevalence settings (<10 % baseline prevalence among SAC) [2, 3]. MDA treatment has mostly focused on SAC since they are thought to be most likely to be infected by *Schistosoma* parasites due, in part, to age-related water contact. Another important factor is that this age group is straightforward to target through school-based deworming and hence a higher MDA coverage can be achieved [5]. However, in high prevalence settings, WHO has also recommended the treatment of adults that are at risk of infection, but progress in getting good treatment coverage in this age group has been poor to date [6].

At present, pre-school age children (pre-SAC) are not treated in public health programmes due to the absence of clinical data on the drug safety and efficacy for young children. It is estimated that nearly 28 million pre-SAC are infected with schistosomes, where the prevalence of infection sometimes surpasses 60% [7]. Clearly, there is a need for a paediatric formulation to achieve morbidity control and elimination. A new PZQ formulation has been developed by the Paediatric Praziquantel Consortium which is a small tablet with an acceptable taste for younger children. After some very encouraging results in the Phase II trials, the Consortium is starting a Phase III study to evaluate the efficacy and safety of this formulation [8].

Recent mathematical model-based results on *S. mansoni*, show that under the current WHO guidelines, it is possible to achieve the WHO goals in low prevalence settings, whereas in moderate prevalence settings, the outcome depends on the baseline prevalence among SAC. The goals are not predicted to be achieved in certain high prevalence settings [9–12]. In such settings, increasing the SAC coverage above 75% and including adult treatment is required to achieve the goals. Alternatively, increasing the treatment frequency to twice a year is also predicted to achieve the WHO goals given good coverage.

The majority of recent transmission modelling for schistosomiasis has focussed on *S. mansoni*, rather than *S. haematobium*, partly due to limited data availability, and because of challenges in interpreting the available data. For both *S. haematobium* and *S. mansoni*, age-stratified pre-treatment surveys show a peak in infection levels amongst children and teenagers [13, 14]. Infection levels, both intensity of infection (as measured by egg output) and prevalence, are generally lower for adults, and this is thought to be partly driven by behaviour [13]. However, for *S. haematobium*, in contrast to *S. mansoni*, a sharp drop in egg intensity and prevalence is seen in adults. It is possible that this is partly due to immunity [15], but it has been challenging to infer immunity parameters and so a behavioural model is a sensible first step.

In this paper, we use an individual-based stochastic model to explore the effect of MDA on *S. haematobium* and examine whether we can achieve the WHO goals of morbidity control and EPHP by using the current guidelines.

## Methods

### The model

We use an individual-based stochastic model which has been developed and published in previous work [14, 16, 17]. Briefly, the deterministic template of the stochastic model is based on sets of partial differential equations which describe the changes over time and age of the mean worm burden, the distribution of parasite numbers within the human host population and of the reservoir of infection defined as the infective cercarial stages in the environment as released by the intermediate snail population. We assume that the parasitic worms are monogamous and that their distribution has a negative binomial form with aggregation parameter *k*.

As worms tend to aggregate more in some individuals than others, we assign a contact rate to each individual person at birth, drawn from a gamma distribution with unit mean and a variance of *1/k*. The total egg contribution to the reservoir from infected humans is governed by the age-dependent worm load, and infection acquisition is assumed to be age-dependent and related to the output of eggs that release miracidia to infect the snail intermediate host, as in previous work [10]. We also assume that the dynamical timescale of the miracidial, cercarial and snail intermediate host, i.e. the reciprocal of their typical respective life spans, are all very fast relative to the life span of the adult worms in the human host (hours and weeks compared with years for the adult worms) such that the equations describing the dynamics of these stages can be collapsed into the equation representing changes in adult worm load in the human host [16].

### Data and parameter fitting methodology

In this paper, we used *S. haematobium* age-intensity of infection profiles from two different communities prior to intensive MDA to fit our model.

**Fig. 2:**
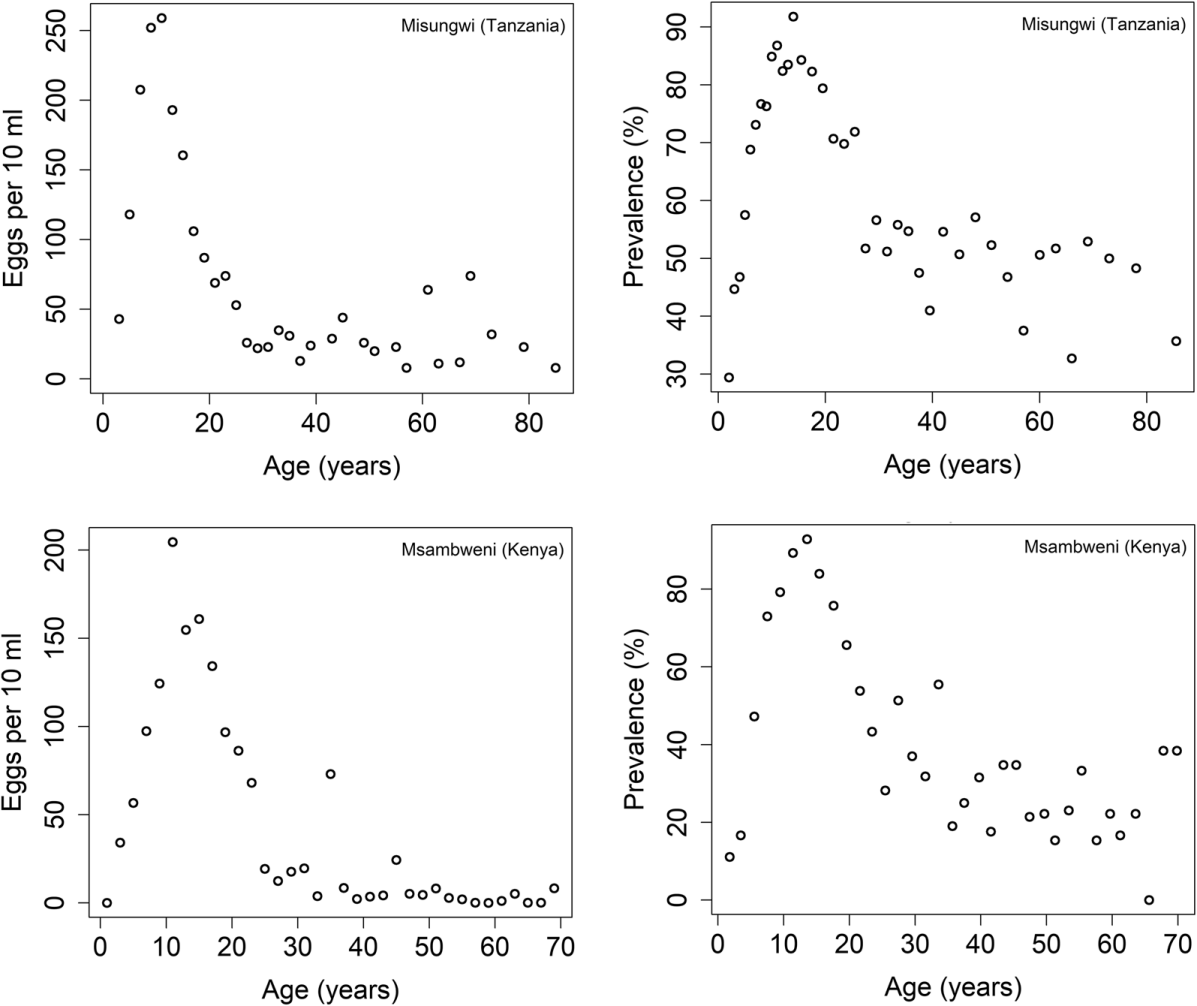
Age-intensity and age-prevalence cross-sectional profiles for *Schistosoma haematobium* in Tanzania (first row) and Kenya (second row) [18, 25]

The first dataset is from a cross-sectional study of 4168 people from the Misungwi area of Tanzania where no MDA had taken place. In this study, age-related mean egg count and prevalence data were recorded for individuals aged 2 to 90 (Fig. 2). For each individual, the egg counts were calculated as the average egg count of two urine samples of 10 ml. The details of sample collection and examination are recorded in reference [13].

The second dataset is from the Msambweni area of Kwale District in Coast Province, Kenya [18, 19] where some very limited and poorly documented MDA had taken place prior to data collection. In this study, age-related egg counts per 10 ml were recorded and the prevalence of morbidity was determined by urine dipstick to detect hematuria [20].

These two datasets are essentially representative of baseline pre-treatment data from two different communities (and timepoints). From Fig. 2, we observe that Misungwi area has a higher intensity of disease than the Msambweni area. Note that Misungwi (Tanzania) data corresponds to a moderate adult burden, while Msambweni (Kenya) dataset corresponds to a low burden in adults. It is clearly of interest to analyse how these different age profiles impact the predictions of MDA impact, particularly, the probability of achieving the WHO goals. Note that stochastic models that take account of chance effects in transmission and human demography, predict probability distributions for a particular outcome of control through MDA.

**Fig. 3:**
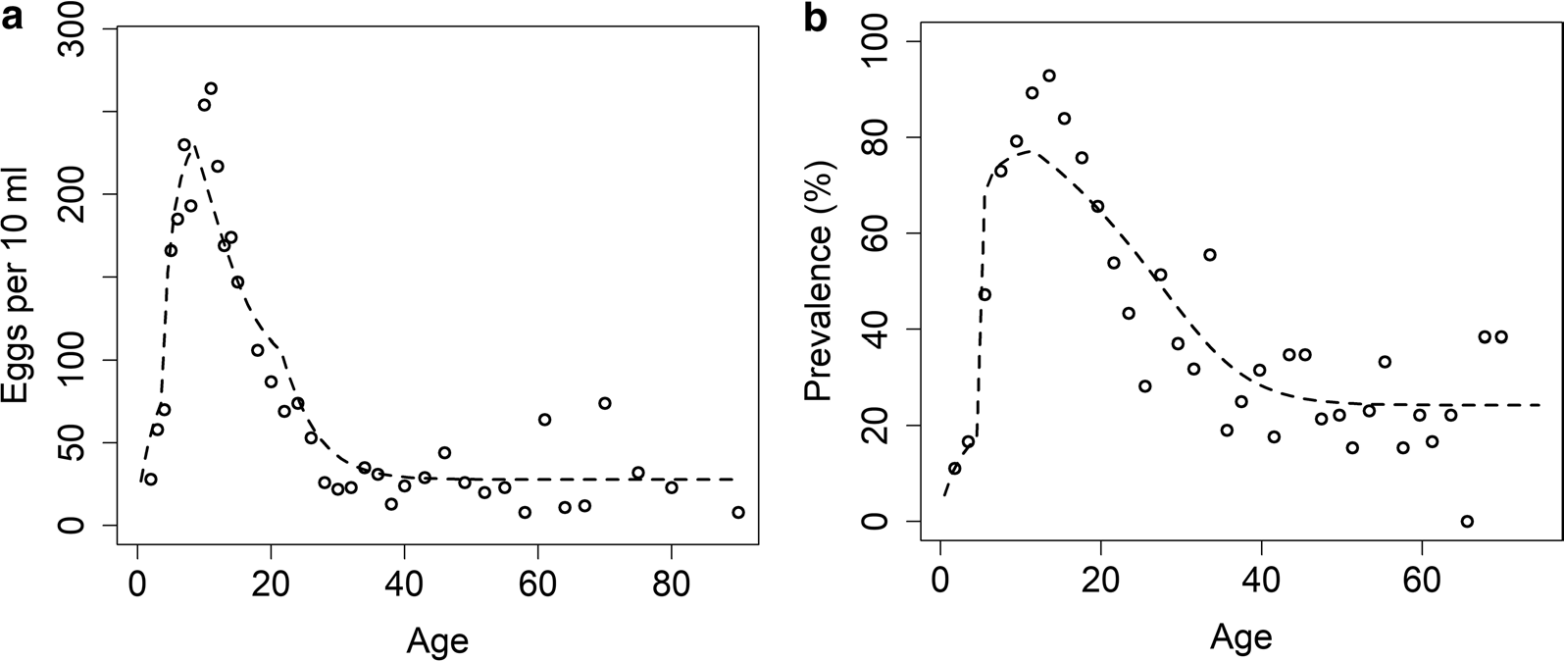
MLE fits as a function of age to (**a**) intensity data for *S. haematobium* (data from Misungwi area (Tanzania) [25]) and (**b**) prevalence data (data from Msambweni area (Kenya) [18])

For both datasets, the maximum likelihood estimates (MLE) for the values of ${R}_{0}$ and age-related contact rates ($\beta$) are obtained by sampling transmission parameters in the deterministic template of the stochastic individual-based model for *S. haematobium* (Figure 3 and Table 1).

**Table 1:** Parameter values for *Schistosoma haematobium* used in making model predictions of MDA impact (some directly estimated from age-intensity or prevalence profiles and some derived from the literature).

Parameter	Value	Source
Population size	500	–
Fecundity	3.6 eggs/female/sample	[26, 27]
Transmission intensity	R_0_ = 2.5	Fitted
Level of aggregation of parasites in host population	Negative binomial, *k* = 0.24	[28]
Adult worm life expectancy	4 years	[16, 17]
Praziquantel drug efficacy	99%	[27, 29]
Age specific contact rates (${\varvec{\beta}}$) for the first dataset [25]	For 0–4, 5–11,12–20, 21+ years of age: 0.17, 1, 0.11, 0.035	Fitted
Contribution to the reservoir by contact age group (${\varvec{\rho}}$) for the first dataset [25]	For 0–4, 5–11,12–20, 21+ years of age: 0.17, 1, 0.11, 0.035	Fitted
Age specific contact rates (${\varvec{\beta}}$) for the second dataset [18]	For 0–4, 5–11, 12+ years of age: 0.0035, 1, 0.0037	Fitted
Contribution to the reservoir by contact age group (${\varvec{\rho}}$) for the second dataset [18]	For 0–4, 5–11, 12+ years of age: 0.0035, 1, 0.0037	Fitted
Prevalence of infection	Percentage of population having > 0 eggs/10 ml	–
Heavy-intensity infection prevalence	Percentage of population having ≥ 50 eggs/10 ml	[2, 30]
Human demography	Based on Kenya’s demographic profile	–

### MDA treatment and model output

Starting with an untreated population, we followed the WHO-recommended guidelines, by treating 75% of SAC with PZQ over a 15-year period. In this study, due to the absence of data from the two studies in Kenya and Tanzania, we have not considered the impact of different patterns of individual adherence to treatment. We assume that treatment is delivered at random at each round.

The treatment frequency depends on the baseline SAC prevalence. In low transmission settings (< 10% baseline prevalence among SAC), MDA occurs once every 3 years, in moderate transmission settings (10–50% baseline prevalence among SAC), MDA occurs every two years and in high transmission settings (≥ 50% baseline prevalence among SAC), MDA occurs every year. To achieve different baseline prevalence, we varied the value of ${R}_{0}$, (reproduction number) where a high (low) value corresponds to a high (low) baseline prevalence.

At the end of the treatment period, we examined the predictions to see from a defined number of simulations runs (300) the probability that the morbidity control goal and EPHP have been achieved, based on the prevalence of heavy intensity infection in SAC. For each treatment strategy, we projected the prevalence of infection, prevalence of heavy intensity infection and the probability of achieving the WHO goals as an average over 300 simulations.

## Results

In this section we present the stochastic model predictions for *S. haematobium* in low, moderate, and high transmission settings.

**Fig. 4:**
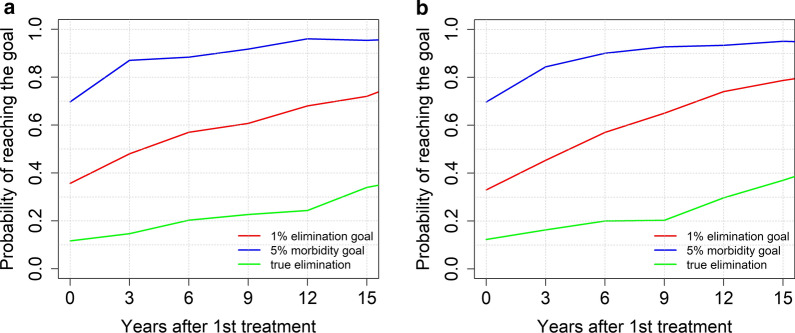
Model projections for school-based (SAC) age group targeted MDA (75% coverage), showing the probability of reaching the WHO goals for moderate burden prevalence in adults (**a**), and low burden prevalence in adults (**b**) in low transmission settings (low R_0_ values)

In **low** transmission settings, treating 75% of SAC every 3 years, can achieve both goals with a high probability (0.95 for morbidity control and 0.75 for elimination as a public health problem), regardless of the age-intensity profile of infection (refer to Fig. 4). In these settings, we can achieve true elimination (overall prevalence reduces to 0%) with a probability of nearly 0.35 after 14 years of MDA.

**Fig. 5:**
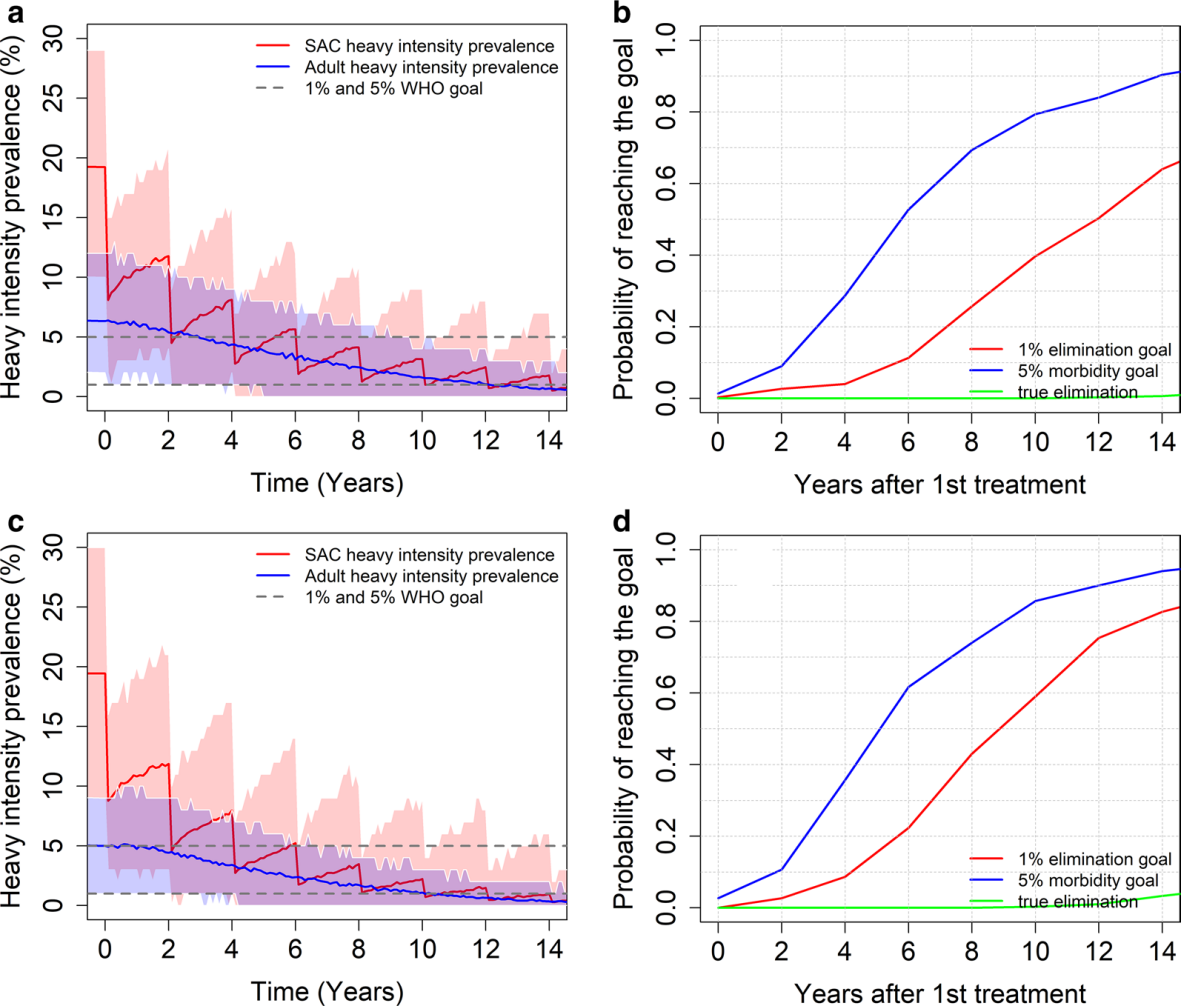
Model projections for school-based MDA (75% coverage), for moderate burden of the intensity of infection in adults (**a**, **b**) and low burden of infection intensity in adults (**c**, **d**). The graphs show the heavy intensity prevalence (**a**, **c**) and the probability of reaching the WHO goals (**b**, **d**) in moderate transmission settings (moderate R_0_ values)

In **moderate** transmission settings, the predictions depend on the burden of infection in adults. The WHO-recommended guidelines can achieve the morbidity control goal for both age-intensity profiles (Kenya and Tanzania) with a range of probabilities of achieving the desired outcome (see Fig. 5). Given a low burden of intensity in adults, the probability of achieving EPHP is nearly 0.85 (see Fig. 5d). Given a moderate burden of intensity in adults, the probability of achieving the elimination as a public health problem is only 0.65 (see Fig. 5b). We can increase this probability (> 0.8) by increasing the coverage for SAC to 90% or increasing the treatment frequency to twice a year.

**Fig. 6:**
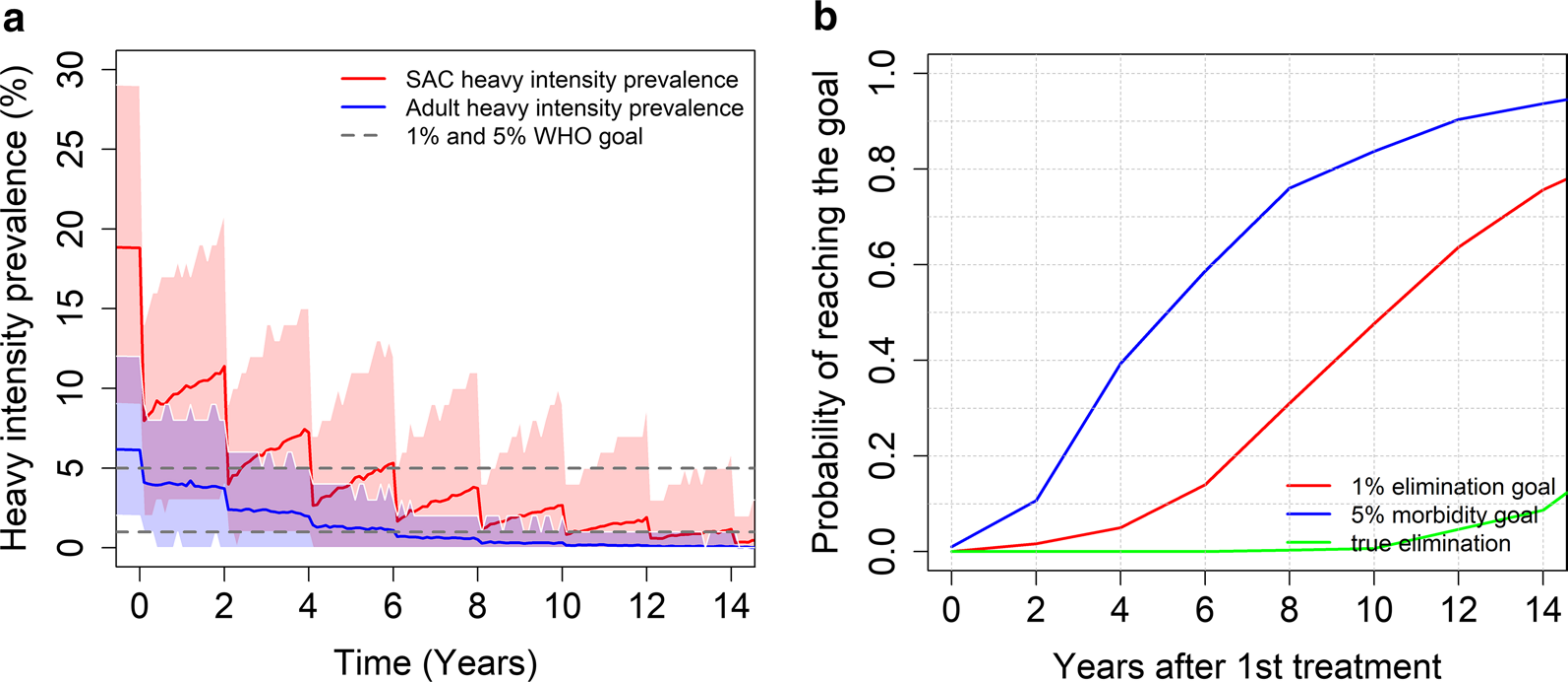
Model projections for community wide MDA (75% SAC + 40% adults coverage), showing (**a**) the prevalence of heavy intensity infection and (**b**) the probability of reaching the WHO goals in moderate transmission settings for moderate burden of the intensity of infection in adults. Treatment is administrated once every two years

Including adults in our treatment programme can also achieve the EPHP goal with a high probability. Treating 75% of SAC and 40% of adults once every two years, can achieve the morbidity control with a probability of 0.95 and the EPHP goal with a probability of nearly 0.79 (see Fig. 6).

**Fig. 7:**
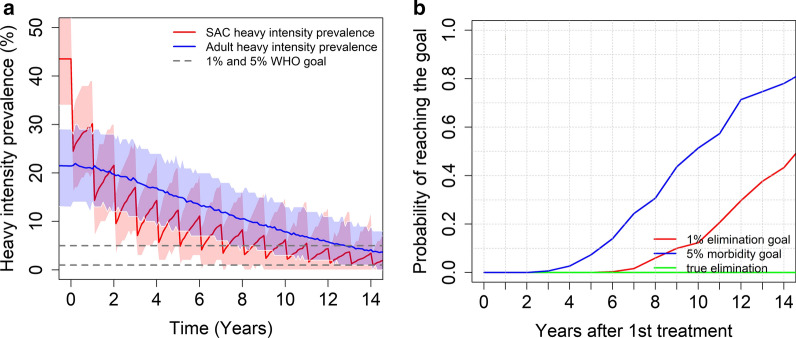
Model projections for school-based MDA (75% coverage), showing (**a**) the prevalence of heavy intensity infections in SAC and adults and (**b**) the probability of reaching the WHO goals in high transmission settings for a moderate burden of infection in adults

In **high** transmission settings, we find that the results depend on the burden of the intensity of infection in adults and the baseline prevalence (the magnitude of R_0_). For a moderate burden of infection intensity in adults, the stochastic model predicts that the morbidity control goal can be achieved within 15 years of starting treatment. On the other hand, the EPHP goal can be achieved with a probability of nearly 0.65 when the baseline SAC prevalence is between 50% and 68% (moderate R_0_ value) and reduces to 0.5 when the baseline SAC prevalence increases to 70% (high R_0_ value) (see Fig. 7).

**Fig. 8:**
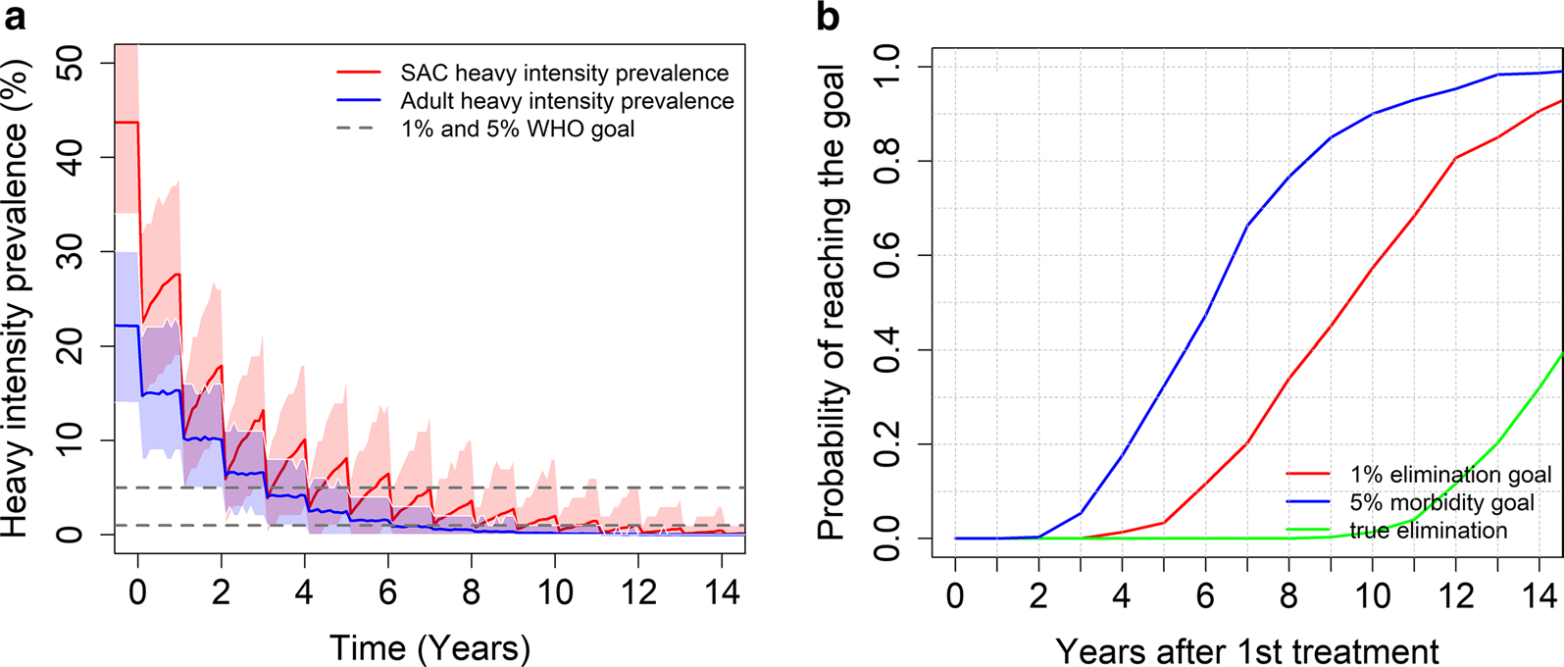
Model projections for community wide MDA (85% SAC + 40% adults coverage), showing (**a**) the prevalence of heavy intensity infections and (**b**) the probability of reaching the WHO goals in high transmission settings for a moderate burden of infection in adults. Treatment is administrated once a year

As in moderate transmission settings, including adults in the MDA programme and increasing the SAC coverage (85% SAC + 40% adults) can achieve the EPHP goal within 15 years of starting treatment (see Fig. 8).

For a low burden of infection intensity in adults, the stochastic model predicts that both WHO goals can be achieved within 15 years of starting treatment for baseline SAC prevalence between 50% and 70%. If we increase the baseline SAC prevalence above 70%, it is necessary to adapt the treatment protocol by increasing SAC/adult coverage and/or increasing treatment frequency to more than once a year. In Table 2, we have summarized the results for different transmission settings (R_0_ values and burden of infection in adults).

**Table 2:** Summary of model projections after following the recommended guidelines set by the WHO and suggestions for programmatic adaptations in cases where the WHO goals are not achieved for *S. haematobium.* We assume the goal is achieved when the probability of achieving it is predicted to be ≥ 0.75 (i.e. 75% plus of the simulations achieve the goal)

Baseline prevalence in SAC	Morbidity goal reached?	Elimination as a public health problem goal reached?	Programmatic adaptation
Low (< 10%)	Low adult burden: Yes; within 2 years Moderate adult burden: Yes; within 2 years	Low adult burden: Yes; within 15 years Moderate adult burden: Yes; within 15 years	Low adult burden: na Moderate adult burden: na
Moderate (10–50%)	Low adult burden: Yes; time depends on the baseline prevalence in SAC	Low adult burden: Yes; time depends on the baseline prevalence in SAC	Low adult burden: na
	Moderate adult burden: Yes; time depends on the baseline prevalence in SAC	Moderate adult burden: Varies depending on scenario	Moderate adult burden: Include adult treatment at 40% coverage
High (≥ 50%)	Low adult burden: Yes; time depends on the baseline prevalence in SAC	Low adult burden: Varies depending on scenario	Increase coverage to 85% for SAC and include adult treatment at 40% coverage
	Moderate adult burden: Yes; time depends on the baseline prevalence in SAC	Moderate adult burden: Not reached	

*na* not required

## Discussion

Using an individual-based stochastic model, we have explored the effect of MDA on *S. haematobium* transmission and determined the probability of achieving the current WHO goals of morbidity control and EPHP. Previous epidemiological analyses have pointed to the importance of collecting adult data on infection intensity as opposed to SAC only data (which is the typical monitoring and evaluation strategy at present) to accurately determine what treatment strategy in terms of coverage by age group and frequency is required to meet the WHO goals [12]. For this reason, we use two different age-intensity profiles of infection prevalence and intensity for *S. haematobium* (data from Misungwi (Kenya) and Msambweni (Tanzania)) with moderate and low adult burdens of infection. For each age category in these epidemiological studies, we have estimated the age-dependent infection rates (see Table 1) by obtaining the MLE parameters for epidemiological data.

Our results highlight the fact that the age-intensity profile has a significant impact on the model predictions of the impact of different MDA treatment strategies. In low transmission settings, both WHO goals for controlling morbidity can be achieved within 15 years of the start of MDA treatment, regardless of the burden of the intensity of infection in adults. In these settings it is possible to also achieve the transmission interruption goal with a probability of 0.35. To improve this figure, higher coverage and/or the treatment of adults is required.

In moderate transmission settings, it is possible to achieve the morbidity control goal, while the probability of achieving the EPHP goal depends on the precise form of the age – intensity of infection profile. For a low burden of infection in adults, it is possible to achieve this goal but for moderate burden of infection in adults, this can only be done if the programme is expanded to include 40% MDA coverage of adults. For high transmission settings, the results depend on the baseline prevalence (the magnitude of R_0_), the precise form of the age-intensity of infection profile and which WHO goal is the main target of the intervention strategy. Given a low to moderate burden of infection in adults, it is very likely that the morbidity control goal can be achieved within 15 years of starting treatment. The EPHP goal can be achieved with a probability of 0.65 for baseline SAC prevalence less than 68% with a moderate burden of infection in adults. However, ideally the treatment of adults should be considered for baseline prevalence higher than 68% to achieve the WHO goal. Similar results are obtained for settings with a low burden of infection in adults. The WHO recommended guidelines can achieve the EPHP goal for baseline infection prevalences less than 70%. However, the treatment of adults must be included and/or treatment frequency increased for baseline prevalences above 70% (high R_0_ values).

These conclusions are in line with previous analyses for *S. mansoni* which also support the collection of data on infection in adults as well as in children to assess correctly what treatment strategy and MDA coverage is required to reach the WHO morbidity control goals. In high transmission settings for *S. haematobium*, our analyses predict that treating adult age groups is also required to meet these goals.

Even though the WHO goals are achieved in most of the epidemiological scenarios relevant for *S. haematobium*, it should be noted that these goals are defined on the basis of the heavy intensity prevalence in SAC and not the overall prevalence in the community. This means that while the heavy intensity prevalence is reduced to below 5% or 1% for the morbidity control and EPHP goal respectively, the overall prevalence might still be high. In these circumstances, stopping MDA treatment after the goals are achieved will result in the bounce-back of infection levels to pre-treatment values in the absence of other changes (e.g. reductions in water contact and improvements in sanitation) due to the remaining reservoir of infection. It should be noted that we have assumed random adherence to treatment at each round of MDA with a defined coverage level and no effect of migration. Hence, our predictions may be on the optimistic side as persistent non-adherers can harbour worms creating the reservoir of infection. This issue can be addressed if data on patterns of individual adherence to treatment are collected. But little attention to date has been paid to this issue in schistosomiasis control programmes.

In future work, it would also be of interest to generalise the parameter estimation approach used here to a fully Bayesian method, where the posterior distribution over the baseline transmission parameters with respect to the data is set as the initial condition to the stochastic individual-based simulation. Such a generalisation would offer insights into the impact that local uncertainty in transmission of *S. haematobium* can have on forecasts for control outcomes.

Other interventions, such as WASH, implementation of snail control, or the possibility of including a vaccine in the treatment programme if one becomes available in the coming years [11, 21] can of course increase the probability of achieving the WHO goals. Ideally, a vaccine is needed due to (i) observed reduced drug efficacy in some worm populations [22, 23] and (ii) stopping treatment after achieving morbidity control or EPHP is likely to lead to resurgence of the disease as infection may remain present in the population [10, 12]. Recent experimental studies on a candidate vaccine against schistosomiasis in a baboon model have shown very encouraging results. However, it is very unlikely that this vaccine will be available before 2030 (even if Phase I, II and III each go smoothly) and hence MDA will continue to be the main form of control for many years to come. In these circumstances, individual-based stochastic models provide health workers with important quantitative tools to assess what MDA programme design is best applied in given epidemiological settings.

## Conclusions

We have found that the probability of achieving the morbidity control and EPHP goals depends on the burden of infection in adults and the baseline SAC prevalence. The current guidelines may achieve the morbidity control goal but to achieve elimination as a public health problem they need to be adapted. We hope this study will provide health workers with important quantitative tools to assess what MDA programme design is best applied in given epidemiological settings.

## Data Availability

All data generated or analyzed during this study are included in this published article.
